# Meal size is a critical driver of weight gain in early childhood

**DOI:** 10.1038/srep28368

**Published:** 2016-06-20

**Authors:** Hayley Syrad, Clare H. Llewellyn, Laura Johnson, David Boniface, Susan A. Jebb, Cornelia H. M. van Jaarsveld, Jane Wardle

**Affiliations:** 1 Health Behaviour Research Centre, Department of Epidemiology and Public Health, University College London, Gower Street, London WC1E 6BT, UK; 2 Centre for Exercise, Nutrition and Health Sciences, School for Policy Studies, University of Bristol, 8 Priory Rd, Bristol BS8 1TZ; 3 Nuffield Department of Primary Care Health Sciences, University of Oxford, Radcliffe Observatory Quarter, Woodstock Road, Oxford OX2 6GG, UK; 4 Department for Health Evidence & Department of Primary and Community Care, Radboud University Medical Center, PO Box 9101, 6500 HB Nijmegen, The Netherlands

## Abstract

Larger serving sizes and more frequent eating episodes have been implicated in the rising prevalence of obesity at a population level. This study examines the relative contributions of meal size and frequency to weight gain in a large sample of British children. Using 3-day diet diaries from 1939 children aged 21 months from the Gemini twin cohort, we assessed prospective associations between meal size, meal frequency and weight gain from two to five years. Separate longitudinal analyses demonstrated that every 10 kcal increase in meal size was associated with 1.5 g/wk or 4% (p = 0.005) faster growth rate, while meal frequency was not independently associated with growth (β = 0.3 g/wk p = 0.20). Including both meal parameters in the model strengthened associations (meal size: β = 2.6 g/wk, p < 0.001; meal frequency: β = 1.0 g/wk, p = 0.001). Taken together, the implication is that meal size promotes faster growth regardless of frequency, but meal frequency has a significant effect only if meal size is assumed to be held constant. Clearer advice on meal size and frequency, especially advice on appropriate meal size, may help prevent excess weight gain.

Obesity rates have risen dramatically over the past 30 years, with increases in childhood obesity provoking particular concern^1^. The causes of this secular trend are likely to be multiple and complex; evidence indicates that, children are now consuming food more often, and in larger amounts at each occasion^2^. However, even in the pervasively ‘obesogenic’ environment, individuals vary considerably in weight, making it important to understand the individual behaviours associated with obesity risk. The behaviours that underlie population trends in weight cannot be assumed to explain individual differences.

Overconsumption might occur through eating too often and/or eating too much each time. There is some evidence for cross-sectional associations between weight and the amount of food consumed per eating occasion. Several studies have shown that adults who are obese tend to consume larger meals^3^,^4^,^5^. In children, one analysis of data from the Continuing Surveys of Food Intakes by Individuals (1994–1998) found that meal size (energy consumed per meal) was associated with weight centile in six to 19 year-olds, but not three to five year-olds^6^; another analysis of the same dataset found a linear association between consumed serving sizes (grams consumed per meal) and weight z-scores in one to two year-olds^7^. There are no longitudinal studies exploring the role of meal size in pediatric weight gain.

There is inconsistent evidence of the relationship between meal frequency and weight. A systematic review found no evidence of an association between eating frequency and weight in adults or children^8^, while a meta-analysis of 11 studies in children and adolescents found an *inverse* association between meal frequency and weight^9^. The only longitudinal analysis in children found that higher eating frequency at nine years was associated with *lower* weight gain over a ten year follow-up^10^. There are no longitudinal studies of meal frequency and weight gain in young children.

No study has examined meal size (energy consumed) and meal frequency in the same sample over the same recording period, making it difficult to determine their relative contribution to excess weight gain or obesity risk. There are no prospective studies in young children. We therefore examined associations between the size and frequency of eating occasions and weight gain, using a longitudinal design, in a large cohort of preschool children. We also examined differences in meal parameters by weight status at baseline to provide insights into differences in eating patterns between overweight and healthy weight children.

## Methods

### Study population

We used data from Gemini; a prospective birth cohort of twins set up to examine early growth. The UK Office for National Statistics asked all families with live twin births in England and Wales between March and December 2007 (N = 6754) if their contact details could be passed to the Gemini research team. 3435 (39%) agreed, and 2402 (70% of those contacted; 36% of all births) consented and completed baseline questionnaires. Gemini is comparable to national twin statistics on sex, gestational age, and birth weight^11^,^12^; but the twins were born earlier and had a lower weight than singletons^12^. Parents provided written informed consent, and the study was approved by the University College London Committee for the Ethics of Non-National Health Service Human Research. The methods were carried out in accordance with the approved guidelines.

### Dietary data collection

At 21 months of age, diet diaries were mailed to families, inviting them to record all foods and drinks consumed by both children over three days (two weekdays and 1 weekend day). Diaries were returned for 2714 children (56.5%). We provided detailed instructions, and portion guides adapted from the British Preschool Food Atlas^13^, to illustrate how to accurately record consumption. Diaries were checked, coded, and linked with British food composition tables^14^ to estimate energy and nutrient intake using *Diet In Nutrients Out*^15^.

‘Meals’ were defined as eating occasions in which food was consumed at a single clock time (and drinks, if consumed at the same time). ‘Meal frequency’ was the number of meals per day, and ‘meal size’ the average energy consumed per meal (total energy consumed in eating occasions/eating frequency), averaged over three days. To estimate whether meal composition differed by weight status at baseline (two years of age), we calculated and compared meal weight (g) and composition (percentage of energy (%E) from protein, carbohydrate and fat), and energy density (kcal/g, with and without drinks included), for normal weight and overweight children.

### Anthropometric measures

We used health visitor records (kept routinely up to two years of age) where possible. From two years families were sent electronic weighing scales (Tanita UK Ltd, Yewsley, UK) and a height chart, and were asked to weigh and measure their children every three months.

The longitudinal analysis used all available weight measurements between two to five years, with weight gain (kg/week) as the primary outcome measure. The cross-sectional analysis used weight at two years as the baseline measure. All children in the UK have a two year health assessment by a health visitor so considerably more weight data were available at this age (n = 1711) compared with 21 months (n = 960). If two year weight was missing it was replaced with the next available weight up to 27 months or the previously available weight after 21 months. Children were classified as overweight or normal weight at baseline using weight Standard Deviations Scores (SDS), calculated using LMS Growth macro for Excel^16^. For the reference population, mean SDS is 0 and the standard deviation 1; SDS > 0 indicates higher weight, and SDS < 0 indicates lower weight compared to reference children of the same age, sex and gestational age. Overweight was classified as a weight SDS > 1.04; above the 85^th^ percentile.

### Demographic and background information

Parents reported child sex, date of birth, birth weight, ethnicity (dichotomised into white and non-white). Parent-held health professional records provided maternal education (dichotomised into lower; no university education, and higher; university education) and gestational age.

### Statistical analyses

**Figure 1:**
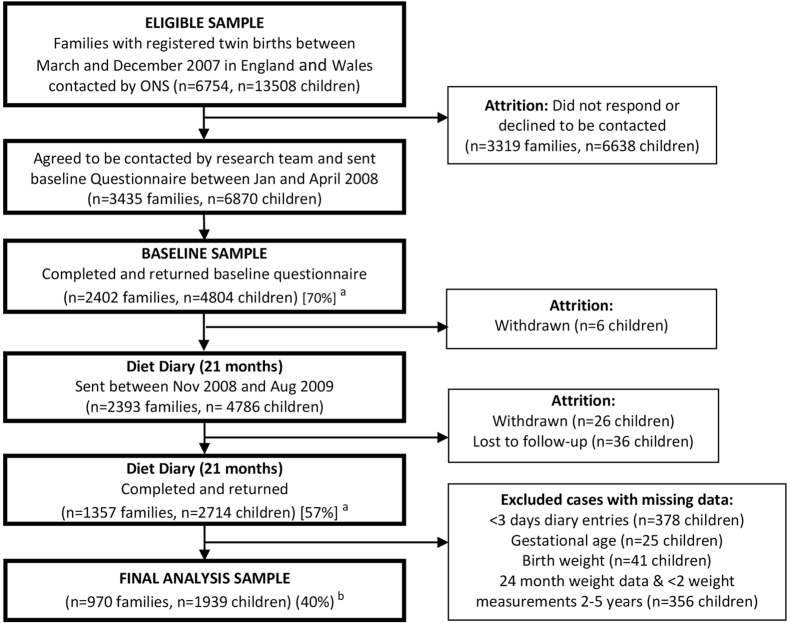
Flow chart of participants from the Gemini study included in final analyses. (**a**) Response rates are given in square brackets [%]. (**b**) Retention rate of cohort for current analyses.

We excluded children without three days of diary entries (n = 378), and those missing weight data at baseline and at least two additional measurements between two to five years (n = 356), gestational age (n = 4), and birth weight (n = 41); leaving 1939 children for analyses; 40% of the baseline sample. Figure 1 shows the flow of included in the analyses. The analysis sample included more mothers of white ethnicity, and were educated to a higher level than non-responders (n = 2865).

Multilevel mixed-effects linear regression explored longitudinal relationships between meal parameters (meal size and frequency) and growth up to five years. All weight measurements of the 1939 children are taken account of. Three-level hierarchical models, accounting for clustering of weight measurements within the child and family, regressed weight on age, sex and relevant dietary measures and their interactions with age, using Stata version 13^17^. The contribution of meal size (per 10 kcals) and meal frequency (per meal) to weekly weight gain (kg and %), in addition to the mean base growth rate of 0.036 kg/wk (coefficient of age in the multi-level model; the growth rate observed in the sample assuming no contribution from dietary intake) was assessed. Two models were run with each meal parameter separately, and a third model was run with both meal parameters included to account of the negative correlation between meal size and frequency (r = −0.55, p < 0.001). An interaction between meal size and frequency was tested by including a product term in the model. Complex Samples General Linear Models (CSGLMs), accounting for clustering of twins within families, examined associations between meal parameters and weight (kg) at baseline (age two years). Two CSGLMs were run with each meal parameter separately; a third model included both meal parameters. CSGLMS also explored mean differences in meal parameters (meal size, frequency and composition) and daily energy intake, by baseline weight status (overweight vs normal weight). Pearson’s correlation established the relationship between meal size (kcals) and meal weight (g) to explore the relationship between meal weight and energy intake.

Birth weight, sex, gestational age, and difference in age between diet diary completion and weight measurement were included as potential confounders. Longitudinal models were additionally adjusted for baseline weight to control for differences in subsequent growth rate driven by earlier weight.

## Results

### Demographics

Sample characteristics are shown in Table 1. There were an equal number of girls (51.2%) and boys and most children were of white ethnic background (95.8%). Children were on average 20.6 months (SD = 1.09) at diary completion, and 24.3 months at baseline weight (two years) measurement. At baseline, the prevalence of overweight/obesity (weight SDS > 1.04) was 17.2%.

### Meal size, meal frequency and weight gain

Table 2 shows that when each meal parameter was assessed separately, variation between children in weight gain from two to five years was partly explained by meal size. For a 10 kcal increase in meal size at 21 months, a child’s growth rate increased by an additional 1.5 g/week, or 4%, above the average growth rate. Meal frequency was not associated with weight gain (ß = 0.3, p = 0.20). However, in the model that included both meal parameters, higher meal frequency was associated with greater weight gain (ß = 1.0; p = 0.001); such that supposing meal sizes were held fixed, each extra meal increased a child’s growth rate by 1 g/week or 2.9%. Furthermore, the association between meal size and weight gain almost doubled (ß = 2.6; p < 0.001) from a 4% increase in weight gain for every 10 kcal increase in meal size, to 7.3%. There was no evidence of interaction between meal size and meal frequency (p = 0.06).

### Meal size, meal frequency and baseline weight and weight status

Table 2 demonstrates that in separate cross-sectional models, meal size was positively associated with weight at baseline (ß = 21; p = 0.002). For every additional 10 kcals consumed per meal, a child weighed 21 g more. Adjusting for meal frequency increased the association between meal size and weight (ß = 33; p < 0.001). Meal frequency was not associated with weight at baseline (ß = 3; p = 0.93); although weak evidence of association emerged when meal size was added to the model (ß = 95; p = 0.02). Results were unchanged using weight SDS as the outcome variable.

Associations between meal parameters and weight status at baseline are shown in Table 3. Overweight children consumed significantly larger meals (190 vs 178 kcals; p = 0.006) and had a greater energy intake (1092 vs 1026 kcals; p < 0.001) than normal weight children, but there was no difference in meal frequency (p = 0.72). The weight of meals (g) was higher in the overweight group (p = 0.002), and there was a significant correlation between meal size (kcals) and meal weight (g) (r = 0.73; p < 0.001). No other meal composition variables (protein, fat, carbohydrate or energy density) were associated with weight status (p-values all > 0.05). Larger meals predicted risk of overweight (OR 1.04; CI 1.01–1.07; p = 0.006); and results were largely unchanged when adjusting for meal frequency. Meal frequency was not associated with risk of overweight (p = 0.72), even when adjusting for meal size (p = 0.18), although the direction of the effect became positive, in line with the continuous associations (Table 4).

## Discussion

This is the first study to explore the relative contributions of meal size and meal frequency on weight gain in young children. In a large sample of twins, larger meal size at 21 months was associated with greater weight gain from two to five years. Meal frequency was not associated with weight gain, except after adjustment for meal size.

Results suggest that young children gain more weight by eating larger amounts at each meal than eating more frequently. For every additional 10 kcals consumed per meal, a child’s growth rate was 4% above the average and the odds of being overweight at baseline was 6% greater.

Although our primary focus was the relationship between meal parameters and weight gain, we also explored associations at baseline; these supported the longitudinal findings. At baseline, meal size, but not meal frequency, was significantly associated with weight (kg). Meal frequency was weakly associated with weight when meal size was included in the model; but it was not associated with risk of overweight, with or without adjustment for meal size. Meal composition (proportions of protein, fat or carbohydrate, or energy density did not differ with weight status (p-values all > 0.05). Overweight children consumed more energy mostly by eating larger quantities of the same composition of food, suggesting a dominant role for meal size, rather than the type of food. The high Pearson’s correlation between meal size (kcals) and meal weight (g) supports this. This is an important issue in an environment where feeding advice often assumes that as long as children are given ‘healthy’ food, they can be left to choose how much to eat. It is sometimes even suggested that parental intrusion into the child’s food quantity decisions could be harmful. Overweight parents for example have been shown to engage in more restrictive feeding behaviours in young children which may result in poorer energy self-regulation and subsequent weight gain^18^. The moderating effect of factors such as parental weight status on children’s eating behaviours and subsequent weight gain is worth further exploration.

Experimental studies show that children consume more when served larger portions^19^,^20^, but there are few recent studies of consumed meal sizes in everyday life. Waxman & Stunkard (1980) observed food intake over four months in a small sample of families (n = 4), and found that obese boys ate substantially larger meals than their normal-weight sibling. Diet composition and meal frequency were not reported^21^. We and others have shown that heavier children exhibit poorer satiety responsiveness than their leaner counterparts^22^,^23^,^24^,^25^,^26^, We have demonstrated that children with poorer satiety responsiveness consume larger meals^27^, placing them at greater risk of excess weight gain. Numerous studies have also shown young children consume more when served larger portions^19^,^28^,^29^,^30^,^31^. Taken together, this might suggest that carers need to guard against ‘over-serving’.

One previous study found no association between weight and eating frequency in one to two year-olds^7^ and a recent meta-analysis found a negative association^9^. In the current study, meal frequency was not associated with weight gain, unless adjusted for meal size. No previous study has included both parameters concurrently, perhaps because information on meal size is typically lacking from questionnaire-based measures of meal frequency^9^. Here we show that meal size was associated with faster growth regardless of meal frequency, suggesting this is a key target for future studies to test public health guidance. Meal frequency only promotes faster growth if meal size remains constant. However, children eating more frequently typically ate smaller meals; thus increased eating frequency *per se* may not be a problem if meal size is reduced accordingly. The inter-play between guidance on meal size and frequency for weight requires further research.

Strengths of this study include detailed diary data, large sample, health professional measured weights for the first two years, and prospective weight data up to five years. Parents weighed and measured their children after two years, which could introduce error, although we previously showed high correlations between researcher- and parent-measured weights (0.83)^32^. Parental compliance with returning weight records reduced over time, but there was an average of six weight measurements per child after two years; with those with less than two measurements excluded from analyses. Our mixed-models analyses took advantage of all available data, and focused on growth rather than point estimates of overweight; however, the fitted model was likely to be biased towards earlier weights. Nevertheless, associations were essentially unchanged after adjusting for weight at baseline.

We did not have information on energy expenditure and were therefore unable to determine the independent contribution of energy intake on growth or explore the impact of under-reporting on meal parameters. Previous research suggests that adjustment for under-reporting could alter associations between meal frequency and weight, although over-reporting has been shown to be more prevalent at younger ages^6^.

Diet diaries were conducted at one time point so there is a lack of data on changes in diet over time. There is currently no consistent method of defining the parameters of eating occasions^33^,^34^,^35^,^36^ and we chose to denote a meal as any ‘eating occasion’ to avoid subjective judgements based on timing or content which could be unreliable for children of this age. An eating occasion included drinks consumed at the same time as food, which might have affected the energy density of the meal. However, meal energy density was not associated with weight or weight status, either with or without drinks included suggesting our definition of a meal was unaffected by the inclusion of drinks.

As with other cohort studies selection bias may have been introduced as the analysis sample consisted of 40% of the initial baseline Gemini sample, and the twin nature of the sample poses questions about generalizability to singletons. However the diets of children in Gemini are comparable to those recorded in a nationally representative sample^37^.

In conclusion, regardless of meal frequency, larger meal sizes contribute to excess weight in early childhood, and higher weight gain over the preschool years. There is a need for further research into how parental feeding practices and infant feeding behaviour may influence one another. Currently there is little guidance to parents on appropriate serving sizes for young children and an analysis of policies to promote healthy portion sizes in the US found this to be a neglected area^38^. More advice on feeding practices, including meal frequency and especially on meal size, may help prevent excess weight gain.

## Additional Information

**How to cite this article**: Syrad, H. *et al.* Meal size is a critical driver of weight gain in early childhood. *Sci. Rep.* **6**, 28368; doi: 10.1038/srep28368 (2016).

## Figures and Tables

**Table 1:** Sample characteristics (n = 1939 children, n = 970 families).

	**Analysis sample n (%) or mean (SD)**
Sex	
Boys	940 (48.5)
Girls	999 (51.5)
Ethnicity	
White	1858 (95.8)
Non-white	81 (4.2)
Maternal education^a^	
Low/intermediate	959 (49.5)
High	980 (50.5)
Age at baseline weight measurement (months)	24.35 (1.02)
Age at diet diary completion (months)	20.58 (0.97)
Gestational age (weeks)	36.20 (2.46)
Weight at birth (kg)	2.46 (0.54)
Weight SDS at birth	−0.55 (0.92)
Weight at baseline (kg)	12.31 (1.44)
Weight SDS at baseline	0.07 (1.03)
Weight status at baseline^b^	
Normal-weight	1606 (82.8)
Overweight/obese	333 (17.2)

Abbreviations: SD, standard deviation; m, months; wk, weeks; kg, kilograms; BMI, body mass index; SDS, standard deviation score.

^a^Maternal education was dichotomised into lower (no university education) and higher (university education).

^b^Weight status at baseline (two years of age) was derived using weight standard deviation scores (SDS). Children were classified as overweight (n = 333) or normal weight (n = 1606) relative to the UK population mean in 1990, for the child’s age, sex, and gestational age^39^. Overweight was classified as weight SDS > 1.04 which equates to scores above the 85^th^ percentile^39^, and normal weight (n = 1606) as SDS< = 1.04.

**Table 2:** Meal parameters, baseline weight and growth from two to five years (n = 1939).

MEAL PARAMETER		Two year weight^a^		Two year weight SDS^a^		Growth rate (g/wk)^b^ (coefficients of interactions with age)		
		**ß (SE)**	**p-value**^c^	**ß (SE)**	**p-value**^d^	**ß (SE)**	**% growth increase**^e^	**p-value**^f^
Meal size (10 kcals per eating occasion)^g^	Separate models	21 (7)	0.002	0.016 (0.005)	0.002	1.5^b^ (0.5)	4.0^c^	0.005
	Mutual adjustment models	33 (8)	<0.001	0.024 (0.062)	<0.001	2.6^b^ (0.6)	7.3^c^	<0.001
Meal frequency (meals per day)	Separate models	3 (35)	0.93	0.001 (0.026)	0.967	0.3 (0.3)	0.9^d^	0.20
	Mutual adjustment models	95 (41)	0.02	0.067 (0.03)	0.03	1.0 (0.3)	2.9^d^	0.001

Abbreviations: ß = unstandardized coefficient, SE = standard error, SDS = Standard Deviation Score.

^a^Analyses have been adjusted for sex, gestational age, birth weight, difference in age between diet diary completion and weight measurement as potential confounders.

^b^Analyses have been adjusted for sex, gestational age, birth weight and weight at 24 months of age as potential confounders. The intra-class correlations at the family and twin levels were 0.39 and 0.47 respectively. At the family level this value represents the between family variance in weight as a proportion of the total variance in weight. At the twin level the value represents the between child variance in weight as a proportion of the total variance in weight over repeated measurement occasions. The random portions included in the longitudinal model were the intercept at the family and intercept and slope of age at the twin levels.

^c^p-value for significance of coefficient: associations between 2 ycoefficient: associations between 2 year weight and meal parameters.

^d^p-value for significance of coefficient: associations between 2 ycoefficient: associations between 2 year weight SDS and meal parameters.

^e^% growth increase in addition to the mean base growth rate (36 g/wk) was calculated by dividing the B coefficient by the mean growth rate (36 g/wk) and multiplying by 100.

^f^p-value for significance of B coefficient: interactions between meal parameters and age.

^g^coefficient: associations between 2 y coefficient has been re-scaled by multiplying by 10 (per 10 kcals); for each 10 kcals increase in meal size a child’s weight at 2 years would be 21 g higher and growth rate would increase by 1.5 g/week in addition to the mean base growth rate (36 kg/wk).

**Table 3:** Meal parameters by weight status^a^ at baseline (two years of age).

**MEAL PARAMETER**	**Full sample (n = 1939)**			**Normal weight (n = 1606)**			**Overweight (n = 333)**			***P*** **value**^b^
	**Mean (SD)**	**Min**	**Max**	**Mean (SD)**	**Min**	**Max**	**Mean (SD)**	**Min**	**Max**	
Meal size (kcals per eating occasion)	180 (49)	59	417	178 (49)	59	417	190 (49)	77	347	<0.001
Meal frequency (meals per day)	5.0 (1.0)	1.7	9.7	5.0 (1.0)	1.7	9.7	5.0 (1.0)	2.7	8.7	0.53
Daily energy intake (kcals per day)	1038 (185)	445	1862	1026 (182)	445	1701	1092 (187)	648	1862	<0.001
Meal composition										
Meal weight (g)	191 (61)	36	401	188 (60)	36	395	205 (65)	75	401	<0.001
Meal energy density (kcal/g)^c^	1.3 (0.4)	0.5	3.2	1.3 (0.4)	0.5	3.2	1.3 (0.4)	0.6	3.0	0.11
Protein per meal (%E)	11.8 (1.8)	6.2	21.1	11.8 (1.7)	6.1	21.1	11.9 (1.7)	8.0	17.3	0.58
Carbohydrate per meal (%E)	54.8 (6.1)	26.9	77.8	54.8 (6.1)	26.9	77.8	54.5 (6.0)	41.3	77.3	0.38
Fat per meal (%E)	33.4 (5.2)	13.3	64.5	33.4 (5.2)	17.4	64.5	33.6(5.0)	13.4	48.9	0.45

Abbreviations: SD, standard deviation; %E, percentage of meal energy; SDS, Standard Deviation Score.

^a^Weight status at baseline (two years of age) was derived using weight standard deviation scores (SDS). Children were classified as overweight (n = 333) or normal weight (n = 1606) relative to the UK population mean in 1990, for the child’s age, sex, and gestational age^39^. Overweight was classified as a weight SDS > 1.04 which equates to scores above the 85^th^ percentile^39^, and normal weight as weight SDS<  = 1.04; below the 85^th^ percentile.

^b^Univariate Complex Samples Linear Regression Models (CSGLMs) tested for significance of mean difference between normal weight and overweight children for each meal parameter; significant differences (p-value < 0.01) are shown in bold.

^c^Results are largely unchanged by calculating energy density of food only (excluding the contribution of drinks to the weight of each meal) (p = 0.84).

**Table 4:** Relative risks of overweight compared to normal weight at baseline (two years of age) according to meal parameters.

**MEAL PARAMETER**	**Model**	**Risk of overweight**^a^ **(n** = **1939)**	
		**OR (95% CI)**	***P*****value**^e^
Meal size (10 kcals per eating occasion)	1^b^	1.05 (1.02; 1.08)	<0.001
	2^c^	1.04 (1.01; 1.07)	0.006
	3^d^	1.06 (1.02; 1.09)	0.001
Meal frequency (meals per day)	1^b^	0.95 (0.82; 1.11)	0.53
	2^c^	0.97 (0.83; 1.14)	0.72
	3^d^	1.13 (0.94; 1.36)	0.18

Abbreviations: OR, Odds Ratio; CI, Confidence Interval.

^a^Weight status at baseline (two years) was derived using weight standard deviation scores (SDS). Children were classified as overweight (n = 333) or normal weight (n = 1606) relative to the UK population mean in 1990, for the child’s age, sex, and gestational age^39^. Overweight was classified as weight SDS > 1.04 which equates to scores above the 85^th^ percentile^39^, and normal weight (n = 1606) as SDS< = 1.04.

^b^Model 1: Univariate complex samples logistic regression analyses tested the odds of being normal weight versus overweight for higher levels of each meal parameter. Models were unadjusted for covariates.

^c^Model 2: Multivariate complex samples logistic regression analyses tested the odds of being normal weight versus overweight for higher levels of each meal parameter. Models were adjusted for sex, gestational age, birth weight, difference between age at diet diary completion and weight measurement.

^d^Model 3: Multivariate complex samples logistic regression analyses tested the odds of being normal weight versus overweight for higher levels of each meal parameter. Models were adjusted for sex, gestational age, birth weight, difference between age at diet diary completion, weight measurement and mutually adjusted for each meal parameter.

^e^p-values in bold represent those <0.01.
